# Academy of Stomatology

**Published:** 1902-10

**Authors:** 

**Affiliations:** Academy of Stomatology


### ACADEMY OF STOMATOLOGY.

A REGULAR meeting of the Academy of Stomatology of Philadelphia was held at its rooms, 1731 Chestnut Street, on the evening of Tuesday, February 25, 1902, the President, Dr. S. B. Luckie, in the chair. A paper entitled "A Peculiar Case of Resorption," written by Dr. Louis Jack, was read by Dr. Charles Jack.

(For Dr. Jack's paper, see page 633.)

### DISCUSSION.

*Dr. E. T. Darby.*—I would say, Mr. President, that Dr. Jack very kindly sent for me to examine this case just prior to filling it. The case was an exceedingly interesting one. It looked, when I first saw it, like a cavity of decay minus any decay. It was irregular in shape and presented the appearance of cavities that we see on labial and buccal surfaces, except that the tooth was white and the dentine was perfectly hard, and there was no indication whatever of a carious condition. I did not see it until after Dr. Jack had pressed the gum-tissue away with cotton. He removed that, and there was a little hemorrhage, which was easily controlled, and then after washing the cavity thoroughly I had a nice opportunity of exploring it, which I did with instruments, by aid of the electric light. It was undoubtedly a very unusual case of resorption. There could not be any possibility of mistaking it, and I do not remember having seen another like it. The doctor said to me that when he first saw the case he supposed that it was a deposit of serumal calculus beneath the gum. When he came to examine it he found the dark zone of discoloration was the cavity itself with the gum-tissue filling in.

It was a set of very beautiful teeth, and the lady took exquisite care of them; there was no indication of calculus on any of the teeth, and a deposit of serumal calculus there would have been very much out of place in her mouth.

*Dr. E. C. Kirk.*—I would like to know whether, before this gum-tissue was pressed aside, there was any relation of the gum-tissue to that cavity, filling in the cavity, or whether a mould was made of the eroded cavity. Have you any data on that point?

*Dr. Charles Jack.*—Yes. The gum-tissue apparently filled a portion of the cavity, as Dr. Darby has stated, but it did not entirely conform to it. There was quite a shelf of enamel over the gingival cavity.

*Dr. Kirk.*—Some years ago a young girl, about fifteen years of age, came to me for dental care, and in examining her mouth I found a gum festoon between the lateral and central incisors of the upper right side hypertrophied and showing indications of hyperæmia. Her teeth were free from caries. In making the examination I turned up a little flap of gum and found a condition which was similar to the one described by Dr. Jack. The little festoon of gum seemed to have embedded itself in the tooth-structure. It had not only brought about an erosion of the relatively soft tissues of the tooth,—*i.e.,* cementum and dentine,—but also of the enamel; it was erosion of the enamel. There was absolutely no residue by carious action in the cavity. I simply regarded it as a resorption of the hard structures of the tooth due to the action of giant-cells or some disordered glandular structure in the little festoon of gum, which was evidently diseased.

We need, I think, a very careful research into the histology of the gingival margin, though some important work has been done by Dr. Black and others. The exact nature of the epithelial structures has not been clearly made out. They have been designated by Dr. Black as glands; they do have something of a glandular structure, but that has been disputed. Dr. Noyes has published something following Dr. Black on this question, but the interesting point in relation to these epithelial cells is that they become the seat of pathological activity, so to speak, and seem to develop a condition which makes it possible for them to excrete an acid fluid. It may be a glandular secretion, or an alteration of the cellular structure, whereby the equivalent of the osteoclast, or giant-cell, is produced. This has a solvent action due to some erosive liquid emitted at the point of contact. We find that type of tissue at the apices of roots in cases of long-continued irritation of the pericementum; we find it at the ends of long bones in gout, or at various other places in the body where there is an irritant to set up a proliferation of leucocytes, which seem to be diverted from their normal function and develop into the multinuclear cells. This case of resorption is the result of a slight irritation of some character, probably situated in the little epithelial structures to which I have referred, and in which the epithelial cells themselves, or the surrounding tissue-cells, have become abnormally developed into the so-called osteoclast and a solvent fluid has been secreted at that point dissolving the tooth-structure.

*Dr. O. E. Inglis.*—At the November meeting I had the pleasure of mentioning a case of a patient in whose mouth four lower incisors had resorbed in a way somewhat similar to that described by Dr. Jack. In that case, however, the resorption was due to a deposit of calculus, which in all probability produced the primary irritation, which eventually brought about a growth of tissue similar to granulation tissue, and that in turn produced a resorption of the roots, not only on the lingual surface, but on the proximal surface.

A specimen was exhibited by Dr. Fellows at that same meeting which I thought had great interest in this connection, because of the fact that it was an exceedingly rare specimen. It may be of interest if I re-describe it. It was a central incisor taken from the mouth of a lady sixty years of age. This central incisor evidently had had a large deposit of secondary dentine in the pulp-canal as the result of age, and that secondary dentine, after resorption of the original dentine, remained in position. The entire apical half of the root was resorbed and the cervical half was entirely hollowed out by the resorbent papilla in such a manner that the cementum completely encircled the area of resorption, while in the centre a column of secondary dentine projected from the pulp-cavity of the crown to the height of the cementum edge. While resorption is often irregular in its action, this case would seem to indicate a superior resistance to acids in cementum and secondary dentine.

*Dr. James Truman.*—My view of the matter is that it is simply a case of erosion. I cannot understand how anything else could accomplish it. I have seen cases not exactly like this, but somewhat similar. I believe it is an external matter entirely. I do not recall whether or not Dr. Darby said that this case had the characteristics of erosion. If it had it could hardly be called a resorption.

*Dr. Darby.*—It was not like any case of erosion I have ever seen. There was no polish and the surface of the cavity was irregular.

*Dr. Charles Jack.*—The cavity was entirely beneath the gingival margin. You could put an explorer into it, but to do that you would have to lift up the soft parts. With a mouth-mirror alone you would not suspect it.

*Dr. Kirk.*—Could a probe be carried beyond the upper margin of the cavity?

*Dr. Charles Jack.*—No; it stopped there.

*Dr. Kirk.*—I would like to say a word on the question of the polish. That is an important part, and we may go astray on it. I believe that this is distinctly an erosive process, but not a case of erosion in the sense in which we generally apply that term.

Now, the question of the polish in the cavity, as to whether it is erosion or whether it is not erosion, is a misleading feature, for whether we have it or not depends upon two things,—the nature of the solvent which is at work and the character of the tooth-structure on which it acts. If we have a solvent action going on very slowly upon a dense tooth-structure, we will have a fine polish. But if we have a rapid acid action upon a tooth-structure not so dense, we have not the polished surface, but one which is more or less chalky in character. It is a clinical manifestation, simply due to the character of tooth-structure and the solvent at work. We have erosion of teeth produced by a variety of solvents and modified by a variety of tooth-structures, and yet they are essentially all erosive processes.

*Dr. Inglis.*—I would like to ask Dr. Kirk whether he would not regard the position in which these hard tissues are being dissolved as having much to do with the presence or absence of polish? Coronal erosions are subject to the abrasive action of the lip, brush, food, saliva currents, etc., which may remove the soluble products of the reaction between the acid from the labial glands and the tooth-structure; whereas, in a place like this, we find in contact with the cavity surface only soft tissue which is quiescent; that is to say, it does not in any way abrade the tooth surface.

*Dr. Joseph Head.*—I might perhaps make some remarks on that subject which would be germane. I was in Chicago last Saturday, and Dr. Black was showing me through the clinic-room and museum, and he showed me specimens of highly polished proximal erosion in which the labial and platine surfaces were perfectly intact. It was a remarkable fact, and I thought it would be germane.

*Dr. Kirk.*—I have made a study of erosions in many ways for a number of years, and I think we have been misled in regarding this disease as in any way affected by mechanical abrasion. It is entirely a distinct disease produced by a different cause. The question of attrition or mechanical abrasion has nothing whatever to do with the character of appearances we see in the true chemical erosion of the teeth or with the nature of the polish. Dr. Head has given a very pointed illustration of the fact where the disorder occurred on the proximal surface of the teeth, and where the polish was characteristic of ordinary erosion.

*Dr. Jeffries.*—Would it be in order to speak of a case of resorption of a permanent root from the contact of the crown of an impacted tooth with it? I have here a specimen of a bicuspid tooth, showing resorption of the root in a manner somewhat similar to that occurring in a deciduous tooth, and caused by the contact of the crown of a supernumerary impacted in the jaw, at right angles with the root. The resorption has caused an exposure of the pulp. (Tooth exhibited.)

I may also state, while the tooth is being exhibited, that I have in my pocket a specimen of the teeth of some Cliff Dwellers of Colorado. They are, of course, anywhere from a thousand to two or three thousand years old. If the members care to look at them, they are at their service. (Teeth exhibited.)

*Dr. Charles Jack.*—The tooth in question was an upper central incisor. The case has completely recovered. The temperature-rate is normal, and the gum has lost all appearance of inflammation, and is now in a normal condition. The question of pulp irritation is the principal one, but the pulp is evidently now in a normal condition.

*The President.*—The next order of business is casual communications and incidents of practice.

*Dr. Inglis.*—It may be of interest, in connection with incidents of practice, and perhaps in connection with this case, inasmuch as it was filled with "archite," to speak of this new cement. I would like to say that at the time the "archite" people sent me the sample I immersed it in strong ammonia and twenty per cent. lactic acid. That was done in the early part of January, about six weeks ago. At the present time there is no disintegration of the "archite." I sent for a box of it at the same time and put a sample, mixed by myself, in the test-fluids, and there has been no change whatever in either of these samples. A day or so afterwards I made two mixes of Harvard zinc phosphate, and allowed them to set for twenty-four hours, then placed them in the same fluids. In twenty-four or forty-eight hours they had both disintegrated. I simply mention that as corroborating their tests.[1]

*Dr. Charles Jack.*—I have tested several different samples of it in acid, ammonia, and water, and I find very little change in any of them. The surface does become roughened, but after polishing away the roughened surface caused by the action of the different fluids and then putting it back I find it does not change. In one case I had it soften very much and to quite a depth, but in all the others—about a dozen—I did not find any change. I have heard from several gentlemen that the different samples differ very much.[2]

*Dr. H. Roberts.*—I tested a sample sent me on a card. I simply dropped it in a bottle of water, and it did not seem to disintegrate as a powder in the bottom of the bottle. When received, it was as hard as anything could possibly be, and be of a composite nature, but after being in the water a short time I could scratch it down; it was considerably harder than chalk, but did not approach any of the phosphate cements in hardness. That was the only sample I experimented with. While it might stand the alkali and acid test, it will not stand the test of Schuylkill water, which test I made.

*Dr. S. H. Guilford.*—I had a little experience with "archite." I received a box when it was first put out, and though I did not fill many teeth with it, I did fill a few. Among other patients so treated was a young boy who had rather a large proximal cavity in an upper bicuspid, containing a disintegrated gutta-percha filling, which was placed a few years before. I replaced it with "archite." That was in December last. He was in the other day, and the cavity was practically empty. I never had anything dissolve out so quickly.

Adjourned.

Otto E. Inglis,
*Editor Academy of Stomatology.*

---

[1] May 8, after four months, all the specimens are visibly affected by the test-fluids. One practical failure has returned.—O. E. D.

[2] Dr. Louis Jack subsequently reported the failure of the "archite" filling by displacement, the cause apparently being some change in the form of the "archite" at its margins. The cavity was afterwards filled with Fellowship amalgam, which remains in good condition, the tooth being comfortable.—Editor.

